# A detection method of the rescue targets in the marine casualty based on improved YOLOv5s

**DOI:** 10.3389/fnbot.2022.1053124

**Published:** 2022-11-16

**Authors:** Jing Bai, Jiacheng Dai, Zhongchao Wang, Shujie Yang

**Affiliations:** School of Marine Engineering Equipment, Zhejiang Ocean University, Zhoushan, China

**Keywords:** YOLOv5s, small object detection, Unmanned Aerial Vehicles (UAVs), lightweight mode, Marine Search and Rescue (SAR)

## Abstract

In recent years, with the deep exploitation of marine resources and the development of maritime transportation, ship collision accidents occur frequently, which leads to the increasingly heavy task of maritime Search and Rescue (SAR). Unmanned Aerial Vehicles (UAVs) have the advantages of flexible maneuvering, robust adaptability and extensive monitoring, which have become an essential means and tool for emergency rescue of maritime accidents. However, the current UAVs-based drowning people detection technology has insufficient detection ability and low precision for small targets in high-altitude images. Moreover, limited by the load capacity, UAVs do not have enough computing power and storage space, resulting in the existing object detection algorithms based on deep learning cannot be directly deployed on UAVs. To solve the two issues mentioned above, this paper proposes a lightweight deep learning detection model based on YOLOv5s, which is used in the SAR task of drowning people of UAVs at sea. First, an extended small object detection layer is added to improve the detection effect of small objects, including the extraction of shallow features, a new feature fusion layer and one more prediction head. Then, the Ghost module and the C3Ghost module are used to replace the Conv module and the C3 module in YOLOv5s, which enable lightweight network improvements that make the model more suitable for deployment on UAVs. The experimental results indicate that the improved model can effectively identify the rescue targets in the marine casualty. Specifically, compared with the original YOLOv5s, the improved model mAP@0.5 value increased by 2.3% and the mAP@0.5:0.95 value increased by 1.1%. Meanwhile, the improved model meets the needs of the lightweight model. Specifically, compared with the original YOLOv5s, the parameters decreased by 44.9%, the model weight size compressed by 39.4%, and Floating Point Operations (FLOPs) reduced by 22.8%.

## Introduction

**FIGURE 1:**
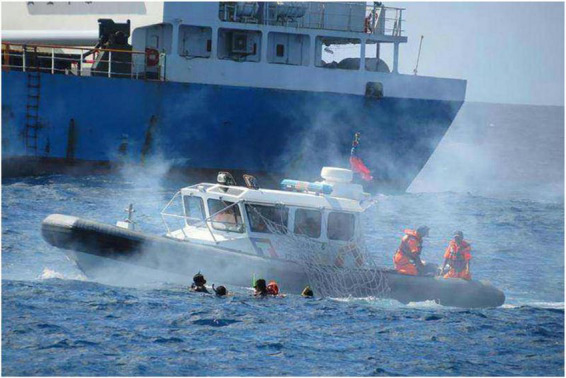
Shipwreck.

Ships are an essential means of transportation for people worldwide to communicate and conduct business. However, while the voyage ships get rich benefits, they also bear the risk of shipwreck (Figure 1). There are many types of shipwrecks, including ships hitting rocks, running aground, colliding with each other, losing control of ships, etc. Shipwreck will cause loss of personnel and property. Marine Search and Rescue (SAR) is crucial to saving the lives of wrecked ships. Traditional maritime SAR relies on a large number of human resources and material resources. An ideal way of modern marine SAR is to be completely unmanned. The Unmanned Aerial Vehicles (UAVs) look for the drowning person and cooperate with the Unmanned Surface Vessels (USVs) to rescue. UAVs reduce unnecessary human risks in rescue operations in severe weather and have the ability to find victims quickly. UAVs can serve as an invaluable modern technology for life-saving emergencies, saving more lives while racing against time in SAR operations at sea and near shore (Yeong et al., 2015).

In recent years, more and more scholars have begun to devote themselves to the application of UAVs in maritime rescue. Ramírez et al. (2011) proposed a coordinated maritime rescue system based on USVs and UAVs. In the proposed system, the USVs benefit from the information provided by the UAVs, which locates the victims faster than the USVs. The USVs are in charge of rescuing the castaways. These two subsystems can work in real-time. The system offered a good foundation for improving automatic object detection effect.

In Leira et al. (2015) proposed a machine vision system that incorporates the use of a thermal imaging camera and onboard processing power. The proposed machine vision system is used to perform real-time object detection, classification, and tracking of objects on the ocean surface.

In Dinnbier et al. (2017) used Gaussian Mixture Model (GMM) and Fourier Transforms for target detection in UAVs maritime SAR. They not only presented a method to combine color analysis and frequency pattern identification, but also dealt with the dynamically changing background through an adaptive algorithm.

Scientific advances in the field of deep neural networks have revolutionized several technical domains, such as image processing (Girshick et al., 2014). In particular, the use of networks based on convolutional topologies brought significant performance gains in the tasks of classification, detection, and automatic image segmentation (Krizhevsky et al., 2017). The networks based on convolutional topologies have also gradually begun to be applied to detection and segmentation tasks at sea, with great success (Cane and Ferryman, 2018).

Target detection algorithms based on deep learning can be divided into two-stage detection algorithms and one-stage detection algorithms. The two-stage detection algorithms select candidate regions and then perform location regression and classification on the candidate regions. Such as Region Convolutional Neural Network (R-CNN) (Girshick et al., 2014), Fast R-CNN (Girshick, 2015), Faster R-CNN (Ren et al., 2015), and Mask R-CNN (He et al., 2017). This type of model has higher detection accuracy but slower detection speed. The one-stage algorithm removes the selection step of the candidate region and directly classifies and regresses the image. Such as the You Only Look Once (YOLO) series (Redmon et al., 2016). This type of model detection speed is faster, but the detection accuracy is slightly lower. Therefore, the YOLO series is more suitable for detecting rescue targets in marine casualties.

Redmon et al. (2016) proposed YOLO for the first time. Drawing on the design idea of Faster R-CNN, they inputted the whole image into the neural network and directly predicted the target position and label at the output stage. Subsequently, YOLOv2 (Redmon and Farhadi, 2017) adopted optimization strategies such as batch normalization, high-resolution classifier and anchor box by Redmon and Farhadi, 2017. YOLOv3 was proposed by Redmon and Farhadi, 2018. The original Darknet-19 network in the feature extraction part is replaced by the Darknet-53 network structure, and the Feature Pyramid Network (FPN) (Lin et al., 2017) structure is used to realize multi-scale detection. Bochkovskiy proposed YOLOv4 (Bochkovskiy et al., 2020) based on Redmon’s research. Through a large number of experiments, it found the best balance among input network resolution, convolution layer number and parameter number. Thereby comprehensive performance of YOLOv4 is improved.

YOLOv5 was proposed by the Ultralytics team in 2020. The author has not published a corresponding academic paper, only the source code has been disclosed, and the website is https://github.com/ultralytics/yolov5. YOLOv5 is faster and more accurate than YOLOv4. There are four versions of the YOLOv5, which are YOLOv5s, YOLOv5m, YOLOv5l, and YOLOv5x. The four models have different widths and depths. Each of the four models has its own merits in terms of performance. The performance of these models is investigated and obtained on the Microsoft Common Objects in Context (MS COCO) test datasets (Lin et al., 2014), as shown in Table 1. It can be concluded from the comparison results that YOLOv5s has the fastest processing speed. Yolov5s is suitable for the application background of this paper and is selected as the benchmark model.

**TABLE 1:** The comparison results of four versions of YOLOv5.

Model	mAP@ 0.5	mAP@ 0.5:0.95	Speed/ms	Params/M	FLOPs/G
YOLOv5s	56.8%	37.4%	6.4	7.2	16.5
YOLOv5m	64.1%	45.4%	8.2	21.2	49.0
YOLOv5l	67.3%	49.0%	10.1	46.5	109.1
YOLOv5x	68.9%	50.7%	12.1	86.7	205.7

The existing models have the problems of low recall rate and high false detection rate when faced with images with many small objects and large-scale changes among various objects. In addition, the existing models have problems of too large volume and too much computation in the process of deploying on UAVs. In view of the above problems, this paper proposes a lightweight detection model for rescue targets in marine casualty based on UAVs image analysis of YOLOv5s, which can significantly reduce the computational complexity, parameter amount, and weight size of the model while maintaining a high model prediction accuracy.

Our main contributions are summarized as follows.

1) In this paper, a data set for the training of the marine rescue model is proposed.
2) This paper adds an extended small object detection layer to improve the detection effect of small objects, including the extraction of shallow features, a new feature fusion layer and one more prediction head.
3) The Ghost module and the C3Ghost enable lightweight network improvements that make the model more suitable for deployment on UAVs.

The remainder of this article is organized as follows. In Section 2, a brief review of the original YOLOv5s model is given. In Section 3, the improved model is proposed. In Section 4, the experiment and result analysis were shown. Finally, the conclusions are summarized in Section 5.

## Introduction to YOLOv5s network

**FIGURE 2:**
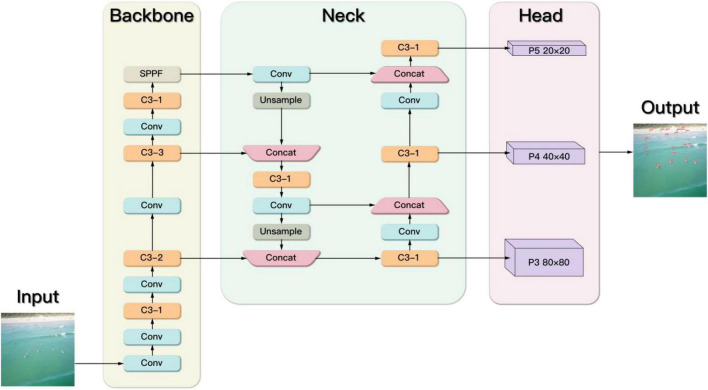
The architecture of YOLOv5s.

YOLOv5s is used as the benchmark model. Each component of YOLOv5s is described below (Figure 2), which includes Input, Backbone, Neck, and Head.

### Input

The input end includes adaptive image scaling and adaptive anchor box calculation. Adaptive image scaling is in the process of image transmission to the network. Many images have different aspect ratios. The common method is to uniformly scale the images to a standard size. Therefore, after scaling and filling, the black borders at both ends are different in size. If there are many black borders filled, it will cause information redundancy and affect the inference speed. However, YOLOv5 employs adaptive image scaling to add minimal black borders. Redundant information is reduced and the inference speed is significantly improved.

During model training, the network outputs the prediction box based on the initial anchor box. The network calculates the gap between the prediction box and the ground truth box. The network obtains the most suitable anchor frame size after backpropagation and parameters iteration. Therefore, the initial anchor size is also a crucial part. The initial anchor size set by YOLOv5 on the MS COCO datasets is shown in Table 2.

**TABLE 2:** Anchor size.

Level	Anchor size
P3	(10,13), (16,30), (33,23)
P4	(30,61), (62,45), (59,119)
P5	(116,90), (156,198), (373,326)

### Backbone

**FIGURE 3:**
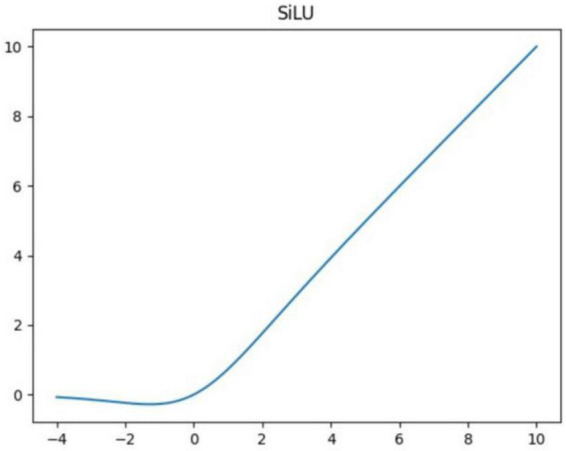
SiLU activation function.

The backbone part is mainly composed of Conv, C3, SPPF, and other modules. The Conv module realizes the output layer by passing the input features through Conv2d function, BatchNorm2d function, and activation function. The activation function uses the Sigmoid-Weighted Linear Units (SiLU) shown in Figure 3.

The SiLU was proposed by Elfwing et al., 2018, which is an approximate function based on reinforcement learning. The SiLU function is computed as Sigmoid multiplied by its input. The activation function *f*(*x*) of the SiLU is given by


(1) f(x)=x⋅σ(x)

where *x* is the input vector, σ(*x*) is the sigmoid function. Sigmoid function is given by


(2) $$\sigma \left(x\right)=\frac{1}{1+e^{-x}}$$

**FIGURE 4:**
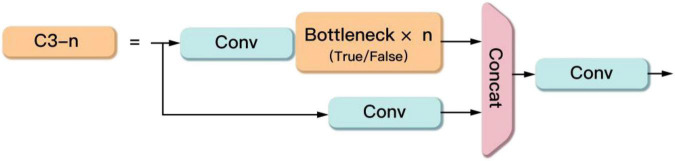
The C3 module.

The C3 module contains three Conv modules and several Bottleneck modules as shown in Figure 4. This module is the main module for learning residual features. Its structure is divided into two branches, one branch passes through a Conv module and several stacked Bottleneck modules in turn, and the other branch passes through only one Conv module. Then the two branches are fused. Finally, the output is obtained through a Conv module.

**FIGURE 5:**
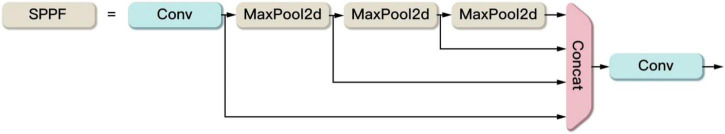
The SPPF module.

Spatial Pyramid Pooling-Fast (SPPF) (Figure 5) is proposed by YOLOv5 authors based on Spatial Pyramid Pooling (SPP) (He et al., 2015), which is much faster than SPP. The SPPF serialize the input through one Conv module and three MaxPool2d layers of 5 × 5 size. Then the four outputs in the serial process are concated. Finally, the output is obtained through a Conv module. Although SPPF pools the feature map multiple times, neither the feature map size nor the number of channels changes, so the four outputs can be fused. The main function of the SPPF module is to extract and fuse high-level features.

### Neck

**FIGURE 6:**
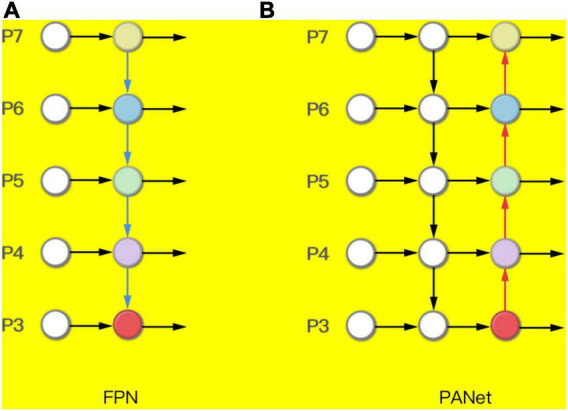
**(A)** Feature pyramid network (FPN) and **(B)** Pyramid Attention Networks (PANet).

The network structure design of the Neck part uses the Pyramid Attention Networks (PANet) (Liu et al., 2018). FPN can fuse features of different resolutions. It can be seen that the FPN in Figure 6A is a top-down route. The high-level strong semantic features are passed down. It can be seen that the PANet in Figure 6B creates bottom-up path enhancement based on FPN to compensate and strengthen the positioning information.

### Head

The loss function in the head part includes the confidence loss *l*_*obj*_, the classification loss *l*_*cls*_, and the bounding box loss *l*_*box*_. The calculation equations are as follows:


(3) $$Loss=l_{obj}+l_{cls}+l_{box}$$

The CIoU Loss (Zheng et al., 2020) function is used to express the bounding box loss *l*_*box*_:


(4) $$l_{box}=L_{CIoU}=1-IoU+\frac{\rho^{2}\left(b,b^{gt}\right)}{c^{2}}+\alpha v$$

where *b* and *b^gt^* denote the central points of the predicted box and ground truth box, *c* is the diagonal length of the smallest enclosing box covering the two boxes, ρ is the Euclidean distance, *v* measures the consistency of the aspect ratio, and α is a positive tradeoff parameter. The formulas are as follows:


(5) $$v=\frac{4}{\pi^{2}}{\left(arctan\frac{\omega^{gt}}{h^{gt}}-arctan\frac{\omega }{h}\right)}^{2}$$

(6) $$\alpha =\frac{v}{\left(1-IoU\right)+v}$$

CIOU Loss takes into account the scale information of the bounding box aspect ratio, which is measured from the angles of aspect ratio, center point distance, and overlap area. Therefore, the speed and accuracy of prediction box regression are greatly improved.

## Improved YOLOv5s network architecture

**FIGURE 7:**
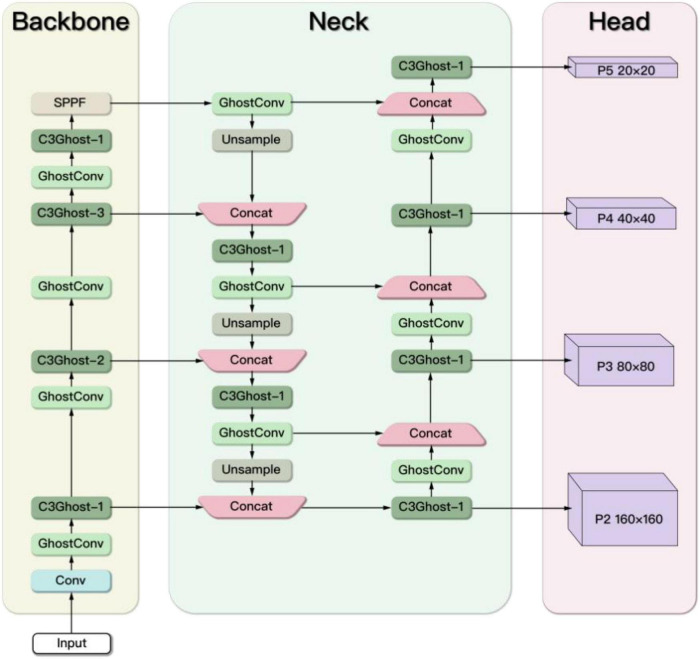
The improved YOLOv5s network architecture.

In order to improve the detection accuracy of the rescue targets in the marine casualty, an extended small object detection layer is added. To achieve a lightweight network design, the Ghost module and the C3Ghost module are used to replace the Conv module and C3 module in the original YOLOv5s. The improved YOLOv5s network architecture is shown in Figure 7.

### The extended small object detection layer

**FIGURE 8:**
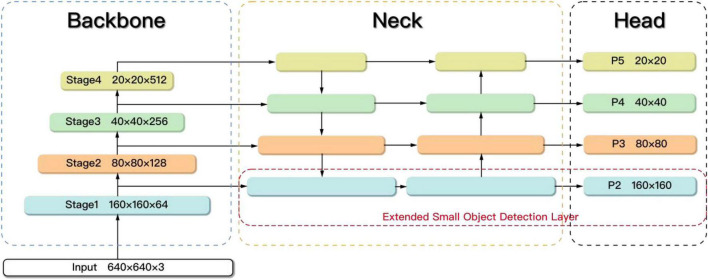
The extended small object detection layer.

The original YOLOv5s model is not good at dealing with small targets. To improve the detection accuracy of small objects, we refer to the method in reference (Zhu et al., 2021; Zhan et al., 2022) and propose an extended small object detection layer as shown in Figure 8. Specifically, the extended small object detection layer consists of the extraction of shallow features, a new feature fusion layer and one more prediction head.

In the Backbone part, the feature maps obtained by different downsampling are set as (Stage1, Stage2, Stage3, and Stage4), which are four times downsampling, eight times downsampling, sixteen times downsampling, and thirty-two times downsampling. The size of the (Stage1, Stage2, Stage3, and Stage4) is 160 × 160, 80 × 80, 40 × 40, and 20 × 20, where Stage1 has the least information loss and the most complete feature information of the small objects. However, only the feature maps corresponding to (Stage2, Stage3, and Stage4) are extracted in the original YOLOv5s, lacking shallow features. It is difficult for the original model to learn the features of small objects. Therefore, the feature map Stage1 obtained by only four times downsampling is extracted in the improved model.

In the Neck part, a new feature fusion layer is added to capture shallow feature information Stage1 from the Backbone. The feature fusion network is changed from three-scale to four-scale, which enhances the learning ability of the network for small targets.

In the Head part of the original network, only prediction heads (P3, P4, and P5) are used, which lead to the loss of small object information. Therefore, the 160 × 160 prediction head P2 is added to receive the low-level, high-resolution feature map from Neck, which is more sensitive to small objects. The resulting new anchor size is shown in Table 3.

**TABLE 3:** New anchor size.

Level	Anchor size
P2	(5,6), (8,14), (15,11)
P3	(10,13), (16,30), (33,23)
P4	(30,61), (62,45), (59,119)
P5	(116,90), (156,198), (373,326)

### Lightweight network design

Limited by the load capacity and computing power of UAVs, the original YOLOv5s model is not suitable for deployment on UAVs. Therefore, the Conv module is replaced by the lightweight Ghost module and the C3 module is replaced by the C3Ghost module to conduct the lightweight network design.

**FIGURE 9:**
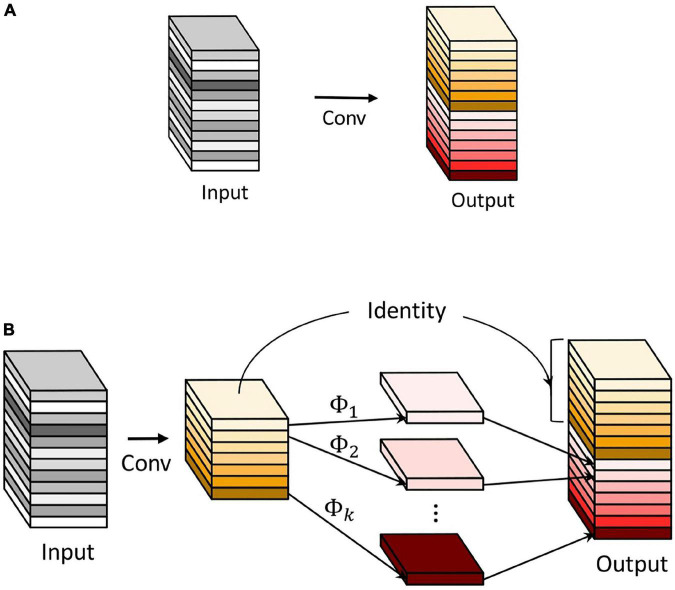
The common convolution and the Ghost module. **(A)** The common convolution. **(B)** The Ghost module.

The Ghost module is a crucial creative module proposed in GhostNet (Han et al., 2020). It has successfully achieved feature maps with few parameters and calculations. The operating principles of the common convolution and the Ghost module are shown in Figure 9. The Ghost module employs common convolution to generate a few intrinsic feature maps. Then cheap operations Φ are utilized to augment the features and increase the channels. The identity mapping is paralleled with linear transformations to preserve the intrinsic feature maps.

Next, the benefits are analyzed between the Ghost module and the common convolution. Floating Point Operations (FLOPs) is used to measure the module complexity. During this common convolution procedure, the required number of FLOPs can be calculated as follows:


(7) $$r_{oc}=n\cdot h^{\prime }\cdot w^{\prime }\cdot c\cdot k\cdot k$$

where *h* × *w* × *c* is the size of input feature map, *h*′ × *w*′ × *n* is the size of output feature map, *k k* is the size of the convolution kernel, ⋅ is multiplication.

During the implementation of the Ghost module, the required number of FLOPs can be calculated as follows:


(8) $$r_{gm}=\frac{n}{s}\cdot h^{\prime }\cdot w^{\prime }\cdot c\cdot k\cdot k+\left(s-1\right)\cdot \frac{n}{s}\cdot h^{\prime }\cdot w^{\prime }\cdot d\cdot d$$

where *d* × *d* refers to the size of the kernel of linear operations (has a similar magnitude as that of *k* × *k*), s refers to the number of cheap transformation operations, and *s*≪*c*.

Comparing the FLOPs of the Ghost module with that of the common convolution, the theoretical ratio can be calculated as follows:


(9) $$r_{s}=\frac{r_{oc}}{r_{gm}}=\frac{n\cdot h^{\prime }\cdot w^{\prime }\cdot c\cdot k\cdot k}{\begin{array}{c} \frac{n}{s}\cdot h^{\prime }\cdot w^{\prime }\cdot c\cdot k\cdot k+\left(s-1\right)\cdot \frac{n}{s}\cdot h^{\prime }\cdot w^{\prime }\cdot d\cdot d \end{array}}\approx \frac{s\cdot c}{s+c-1}=s$$

From formula (9), it can be seen that the FLOPs of the common convolution are approximately *s* times of that of the Ghost module. The statistic of the parameter amount is similar to FLOPs and can also be approximated to *s* times. Apparently, the module cannot only effectively save computing resources, but also reduce the number of parameters.

**FIGURE 10:**
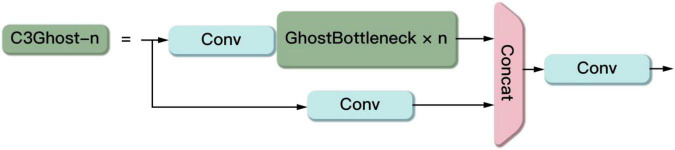
The structure of the C3Ghost.

**FIGURE 11:**
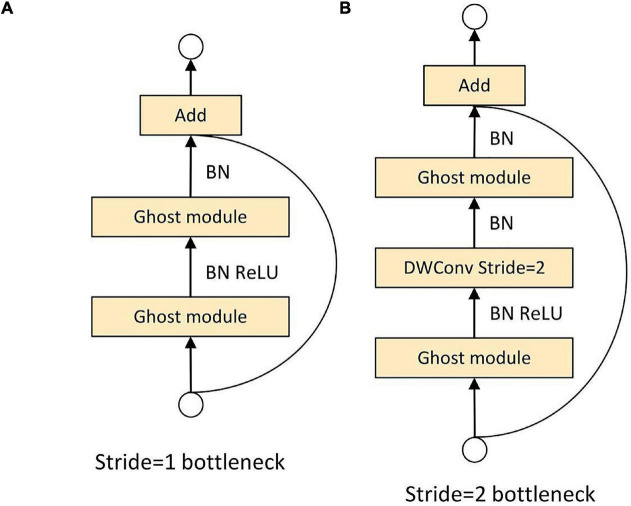
Ghost bottleneck. **(A)** Ghost bottleneck with stride = 1. **(B)** Ghost bottleneck with stride = 2.

The C3Ghost module mainly consists of a Ghost Bottleneck structure and three Conv modules in Figure 10. The Ghost Bottleneck built by taking advantage of the ghost module is shown in Figure 11. When the stride of the Ghost Bottleneck is set to one, it is composed by two stacked Ghost modules. The first Ghost module is an extension layer, which increases the number of channels and increases the feature dimension. The second Ghost module reduces the number of channels and feature dimensions, which compress the network model while ensuring the same number of input and output channels. Finally the inputs and outputs of these two Ghost modules are connected. When the stride of Ghost Bottleneck is set to two, a Ghost module is used to perform feature extraction on the input feature layer. Then a depthwise convolution is used to compress the height and width of the feature layer. Then the second Ghost module is used for feature extraction. Finally the input and output are connected.

## Experiment and result analysis

### Datasets preparing

**FIGURE 12:**
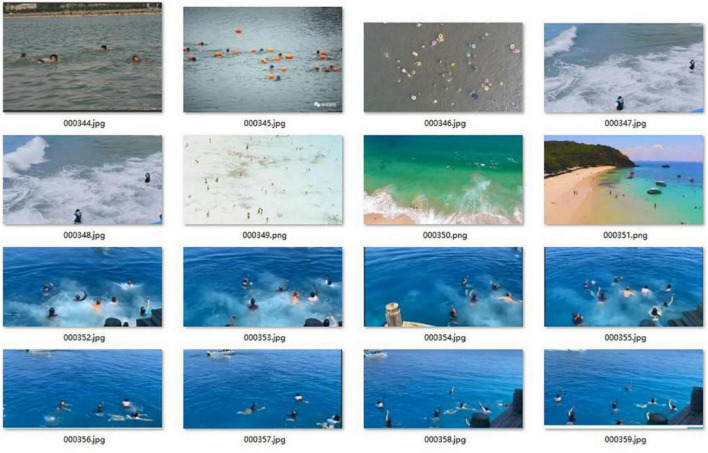
Some pictures of the datasets.

The improved model is used to identify the drowning person in the maritime SAR. However, there is no relevant public datasets. We have collected a large number of pictures and videos of people’s activities in the sea. These data are mainly from the Internet and our seaside aerial photography. For the video data, we adopt the production strategy of extracting one frame every five frames. Some datasets pictures are shown in Figure 12.

**FIGURE 13:**
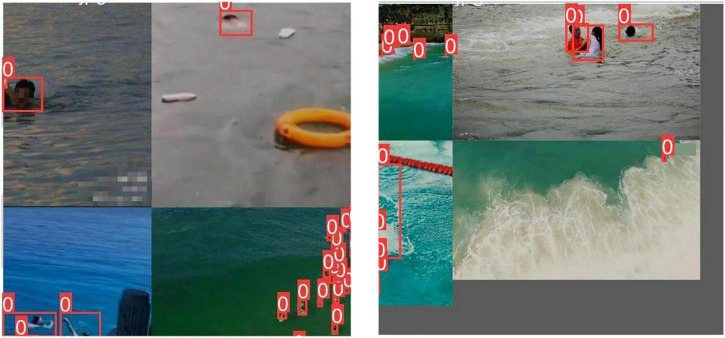
Mosaic data augmentation.

The datasets contain 1,700 original pictures, including 11,880 target objects. The datasets are divided into training set and verification set according to the ratio of 8:2. LabelImg labeling tool is used to calibrate the personnel in each image manually. Mosaic data augmentation (Figure 13) is to crop four pictures and take a part of each and mix them into a new picture. Both the length and width of the cropping position are generated randomly. Mosaic data augmentation not only expands the datasets but also improves the detection effect of small targets.

### Measurement indicators

Some typical performance measurement indicators are used to evaluate the performance of the improved model, including P (Precision), R (Recall), mAP (mean Average Precision), parameter quantity, FLOPs, and model weight size. Before introducing the above indicators, we must present the following basic concepts. IoU (Intersection over Union) refers to the intersection of the prediction box (A) and ground truth box (B) divided by the union of the two. Its formula is:


(10) $$IoU=\frac{A\cap B}{A\cup B}$$

*TP* represents the number of IoU greater than the set threshold, which is calculated only once for the same ground truth box; *FP* represents the sum of IoU less than the set threshold or the number of redundant prediction frames detected for the same real frame; and *FN* represents the number of real frames not detected.

P (Precision) is the correct proportion of all the targets predicted by the model. Precision is calculated by


(11) $$P=\frac{TP}{TP+FP}$$

R (Recall) is the correct proportion of model prediction in all marked targets. Recall is calculated by


(12) $$R=\frac{TP}{TP+FN}$$

AP (Average Precision) is the area enclosed by the curve with recall as the horizontal axis and precision as the vertical axis. The average precision is calculated by


(13) $$AP=\int_{0}^{1}P\left(R\right)dR$$

mAP (mean Average Precision) is used to measure the recognition accuracy, which is the average of all categories of AP . The mean Average Precision is calculated by


(14) $$mAP=\frac{\sum AP}{n}$$

mAP@0.5 represents mAP when IoU is 0.5. mAP@0.5:0.95 represents the average mAP (0.5, 0.55, 0.6, 0.65, 0.7, 0.75, 0.8, 0.85, 0.9, and 0.95) on different IoU thresholds (from 0.5 to 0.95, in steps of 0.05).

### Model training

The specific configurations are provided in Table 4.

**TABLE 4:** Configurations of the experimental platform.

Parameter	Configuration
Programming language	Python 3.8
GPU accelerated environment	CUDA 10.1
Central Processing Unit (CPU)	Intel(R)Core(TM)i9-11900K@ 3.50GHz
Random Access Memory (RAM)	32GB
Graphic Processing Unit (GPU)	NVIDIA GeForce GTX3080Ti

During the training process, the epoch of model training is set to 300, the batch size is set to 8, the learning rate is dynamically adjusted and optimized by the Stochastic Gradient Descent (SGD) optimizer, and the initial learning rate is 0.01. The specific parameter settings of the model are shown in Table 5.

**TABLE 5:** Model training parameters.

Parameter	Value
Input size	640 × 640 ×3
Epochs	300
Batch size	8
Optimizer	SGD
Initial learning rate	0.01

**FIGURE 14:**
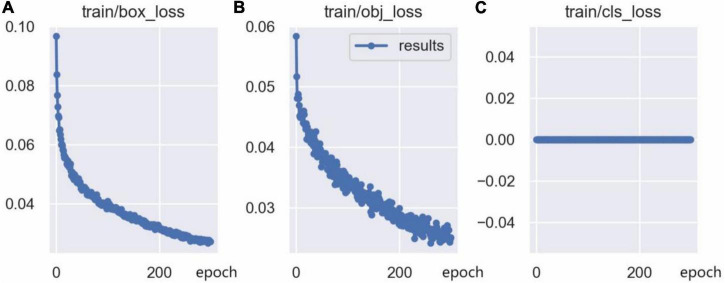
Training loss curve: **(A)** Bounding box loss (CIoU) curve, **(B)** confidence loss curve, **(C)** classification loss curve.

**FIGURE 15:**
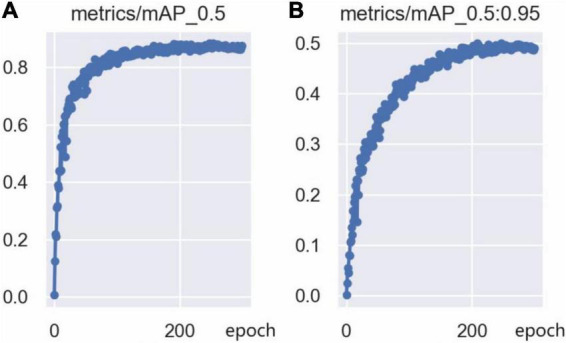
The mAP curve: **(A)** mAP@0.5 curve, **(B)** mAP@0.5:0.95 curve.

The training loss curves are shown in Figure 14, including the bounding box loss curve, the confidence loss curve, and the classification loss curve. The bounding box loss curve and the confidence loss curve indicate that the loss value decreased rapidly in the first 50 epochs of network training, and tends to be stable after 250 epochs of training. Since there is only one class (person) in this model, the classification loss curve remains at 0. Furthermore, the mAP@0.5 curve and mAP@0.5:0.95 curve are shown in Figure 15, which indicates that the model is well trained without overfitting.

### Ablation studies

In order to explore the effect of the two improved parts on the model, we conducted ablation experiments as shown in Table 6. The “YOLOv5s+P2” model represents the model after adding an extended small object detection layer. The computational cost of this model has grown exaggeratedly from 15.8 to 23.4. But at the same time, the detection accuracy is also significantly improved. The “YOLOv5s+P2+Ghost” model represents the model after using the Ghost module and the C3Ghost module based on the above model. The detection accuracy is slightly reduced, but still better than the original YOLOv5s model. The number of parameters decreased from 7352040 to 3860736. The FLOPs dropped from 23.4 to 12.2. The lightweight effect is remarkable.

**TABLE 6:** Ablation study.

Model	mAP@0.5	mAP@0.5:0.95	Parameters	Weight size/M	FLOPs/G
YOLOv5s	86.4%	55.0%	7,012,822	13.7	15.8
YOLOv5s+P2	88.9%	57.9%	7,352,040	14.9	23.4
YOLOv5s+P2+Ghost	88.7%	56.1%	3,860,736	8.3	12.2

### The comparison results with related methods

In order to prove the effectiveness and superiority of the improved model, the improved model is compared with the original YOLOv5s, the mainstream lightweight networks YOLOv4-tiny and YOLOv3-tiny. The results are shown in Table 7. It can be seen in Table 7 that compared with the original YOLOv5s, the improved model mAP@0.5 value increased by 2.3%, the map@0.5:0.95 value increased by 1.1%, the parameters decreased by 44.9%, the model weight size compressed by 39.4%, and FLOPs reduced by 22.8%. Compared with the experimental results of other models in the table, it can be seen that the improved model has the highest detection accuracy, the minimum amount of training parameters, the minimum model weight and the minimum amount of calculation, which is conducive to the deployment of this model on UAVs. It can be concluded that this method has advantages over other mainstream lightweight networks.

**TABLE 7:** The comparison results with related methods.

Model	mAP@0.5	mAP@0.5:0.95	Parameters	Weight size/M	FLOPs/G
YOLOv3-tiny	79.8%	41.6%	8,666,692	16.6	12.9
YOLOv4-tiny	81.5%	46.1%	5,878,736	11.3	16.2
YOLOv5s	86.4%	55.0%	7,012,822	13.7	15.8
Ours	88.7%	56.1%	3,860,736	8.3	12.2

### Visualization result

**FIGURE 16:**
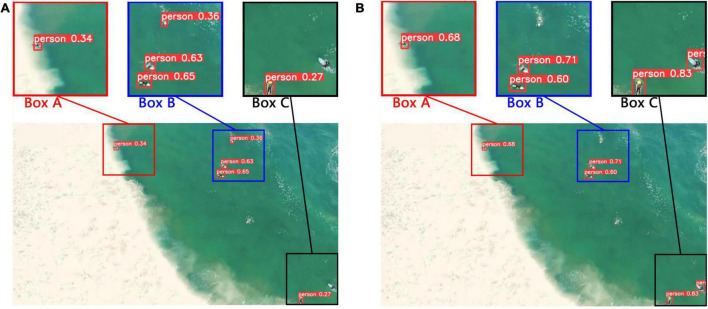
Comparison of visualization results before and after model improvement. **(A)** Detection result of the original YOLOv5s model. **(B)** Detection result of the improved model.

In order to further prove the effectiveness and superiority of our model. Some visualization results of the original YOLOv5s model and the improved model on the test set. See Figure 16 for the practical effect.

Figure 16A shows the detection results of the original YOLOv5s model, and Figure 16B shows the detection results of the improved model. Box A, Box B, and Box C are partial magnifications of the test picture. First of all, by comparing the Box A of Figure 16A and that of Figure 16B, the confidence of the improved model for small object detection is significantly improved. In addition, comparing Box B in Figure 16A and that of Figure 16B, the original YOLOv5s model misjudged the white spray as a person, which led to false detection. However, the improved model has no error detection. Finally, comparing Box C in Figure 16A and that of Figure 16B, the original YOLOv5s model has an omission. However, both people were detected by the improved model. Therefore, it can be concluded that the detection effect of the improved model for small targets is greatly enhanced.

## Conclusion

Due to the target scale in the perspective of the UAVs is small, and these small objects bring difficulties to detection. In addition, due to the limitations of UAVs’ computing power and storage space, UAVs need to carry a model with small weight, few parameters, low computational complexity, and easy deployment. In order to solve the two issues mentioned above, this paper proposes a lightweight detection model for rescue targets in marine casualty based on UAVs image analysis of YOLOv5s. In this paper, the datasets for the training of marine rescue model is proposed. Then, an extended small object detection layer is added to the original YOLOv5s. In the end, the Ghost module and the C3Ghost module are used to replace the Conv module and the C3 module in YOLOv5s, which achieve lightweight network design. It can maintain high model prediction accuracy and significantly reduce the volume of the model. Compared with other mainstream models, the experimental results indicate that the improved model can effectively identify the drowning person and meets the requirements of the lightweight embedded model. This improved model is easier to deploy on UAVs to carry out maritime rescue tasks, and has certain social value.

Although the improved YOLOv5s network has achieved good results in detecting drowning persons, the detection accuracy still needs to be improved. According to our observation, the application background also has the problem of mutual occlusion caused by too dense small targets. In future research, the network model structure will be further optimized to improve the network performance.

## Data availability statement

The raw data supporting the conclusions of this article will be made available by the authors, without undue reservation.

## Author contributions

JB and ZW contributed to the conception and design of the study. JB and JD organized the database. JB performed the statistical analysis and wrote the first draft of the manuscript. JB and SY wrote sections of the manuscript. All authors contributed to manuscript revision, read, and approved the submitted version.
